# 

**Published:** 1897-08

**Authors:** 


### CONTRIBUTORS TO VOLUME XXXVII.

AUGUST, 1897—JULY, 1898.

---

| | |
|---|---|
| Angell, Edward B. | Rochester. |
| Becker, Tracy C. | Buffalo. |
| Blume, Frederick | Allegheny. |
| Brown, J C. | Smethport, Pa. |
| Carpenter, Thomas B. | Buffalo. |
| Clemesha, J. C. | Buffalo. |
| Cott, George F. | Buffalo. |
| Creveling, Joseph P. | Auburn. |
| Crockett, Montgomery A. | Buffalo. |
| Cumston, Charles Greene | Boston. |
| Daggett, Byron H. | Buffalo. |
| Deaver, John B. | Philadelphia. |
| Dowd, J. Henry | Buffalo. |
| Dennis, John | Rochester. |
| Durand, C. Ferdinand | Buffalo. |
| Elsner, S. L. | Rochester |
| Frederick, Carlton C. | Buffalo. |
| Fronczak, Francis E. | Buffalo. |
| Frye, Maud Josephine | Buffalo. |
| Goldberg, Sigmund | Buffalo. |
| Guillemont, F. | Niagara Falls. |
| Henckell, A. W. | Buffalo. |
| Himmelsbach, George A. | Buffalo. |
| Houghton, E. M. | Detroit. |
| House, William | Buffalo. |
| Howe, Lucien | Buffalo. |
| Hubbell, Alvin A. | Buffalo. |
| Jacobson, Nathan | Syracuse. |
| Jones, Frank A. | Rochester. |
| Kiepe, Edward J. | Buffalo. |
| Kinnear, Beverly O. | New York. |
| Krauss, William C. | Buffalo. |
| Lichty, John A. | Clifton Springs. |
| Loveland, B. C. | Clifton Springs. |
| Lytle, Albert T. | Buffalo. |
| Miller, John A. | Buffalo. |
| Murray, C. A. | Van Etten. |
| Orr, C. R. | Buffalo. |
| Parmenter, John | Buffalo. |
| Potter, William Warren | Buffalo. |
| Preiss, Frederick | Buffalo. |
| Pryor, John H. | Buffalo. |
| Putnam, James Wright | Buffalo. |

Ringueberg, Eugene N S. . . . . . . . . . . . . . . . . . . . . . Lockport.
Roe, John O. . . . . . . . . . . . . . . . . . . . . . . . . . . . . Rochester.
Satterlee, Richard H. . . . . . . . . . . . . . . . . . . . . . . . . Buffalo.
Schröter, L. . . . . . . . . . . . . . . . . . . . . . . . . . . . . Buffalo.
Sefton, Frederick . . . . . . . . . . . . . . . . . . . . . . . . . . Auburn.
Smith, Eugene A. . . . . . . . . . . . . . . . . . . . . . . . . . . Buffalo.
Snow, Sargent F. . . . . . . . . . . . . . . . . . . . . . . . . . . Syracuse.
Stanbro, F. H. . . . . . . . . . . . . . . . . . . . . . . . . . . . Springville.
Stephenson, F. H. . . . . . . . . . . . . . . . . . . . . . . . . . Syracuse.
Suiter, A. Walter . . . . . . . . . . . . . . . . . . . . . . . . . Herkimer.
Trowbridge, Grosvenor R. . . . . . . . . . . . . . . . . . . . . . . Buffalo.
Warren, J. Collins . . . . . . . . . . . . . . . . . . . . . . . . . Boston.
Wende, Ernest . . . . . . . . . . . . . . . . . . . . . . . . . . . Buffalo.
Wende, Grover William . . . . . . . . . . . . . . . . . . . . . . . Buffalo.
Wenning, William H. . . . . . . . . . . . . . . . . . . . . . . . . Cincinnati.
Williams, Herbert U. . . . . . . . . . . . . . . . . . . . . . . . . Buffalo.
Wilson, Nelson W. . . . . . . . . . . . . . . . . . . . . . . . . . Buffalo.
Ullman, Julius . . . . . . . . . . . . . . . . . . . . . . . . . . . Buffalo
American Association of Obstetricians and Gynecologists.
Buffalo Academy of Medicine.
Medical Association of Central New York.
Medical Society County of Chautauqua.
Medical Society County of Erie.
Medical Society County of Herkimer.
Niagara Falls Academy of Medicine.
Southern Surgical and Gynecological Association.

### BOOKS RECEIVED.

A Text-Book of Diseases of Women. By Charles B. Penrose, M. D., Ph. D., Professor of Gynecology in the University of Pennsylvania; Surgeon to the Gynecean Hospital, Philadelphia. Octavo, pp. 529. Price, $3.50, net. Illustrated. Philadelphia: W. B. Saunders, 925 Walnut street. 1897.

System of Diseases of the Eye. By American, British, Dutch, French, German and Spanish authors. Edited by William F. Norris, A. M., M. D., and Charles A. Oliver, A. M., M. D., of Philadelphia, Pa. Volume II. Examination of the Eye, School Hygiene, Statistics of Blindness and Antisepsis. Octavo, pp. ix.—556. With thirteen full-page plates and 214 text illustrations. Philadelphia: J. B. Lippincott Co. 1897.

Text-Book on Mental Diseases. By Theo. H. Kellogg, A. M., M. D., Late Medical Superintendent of Willard State Hospital, etc., etc. Octavo, 792 pages; illustrated by original sphygmographic tracings and photographs of the different types of mental disorder. Extra muslin, $6.00.

Eye-strain in Health and Disease. With Special Reference to the Amelioration or Cure of Chronic Nervous Derangements Without the Aid of Drugs. By Ambrose L. Ranney, A. M., M. D., Author of Lectures on Nervous Diseases, The Applied Anatomy of the Nervous System, etc., etc.; Late Professor of Nervous Diseases in the Medical Department of the University of Vermont and of the Anatomy of the Nervous System in the New York Post-Graduate Medical School, etc. Illustrated with thirty-eight wood-cuts. One volume royal octavo, pages, viii.—321. Extra cloth, beveled edges, $2.00, net. Philadelphia: The F. A. Davis Co., Publishers, 1914 and 1916 Cherry street. New York: 117 W. Forty-second street. Chicago: 9 Lakeside Building.

Flint's Medical and Surgical Directory of the United States and Canada. Issued annually. 1897. Compiled by A. L. Chatterton. New York: J. B. Flint & Co., 104 Fulton street. 1897.

A System of Practical Medicine. By American authors. Edited by Alfred Lee Loomis, M. D., Late Professor of Pathology and Practical Medicine in the New York University, and William Gilman Thompson, M. D., Professor of Materia Medica, Therapeutics and Clinical Medicine in the New York University. To be completed in four imperial octavo volumes, containing from 900 to 1,000 pages each; fully illustrated in colors and in black. Volume I. Infectious Diseases. Just ready. Volume II. Diseases of the Respiratory and Circulatory Systems and of the Blood, Kidneys and Genito-urinary Organs. Just ready. Volume III. Diseases of the Digestive System, of the Liver, Spleen, Pancreas and other Glands; Gout, Rheumatism, Diabetes and other Constitutional Diseases. In Press. Volume IV. Diseases of the Nervous System and of the Muscles; Diseases of doubtful origin, Insolation, Addison's Disease, etc. In active preparation. For sale by subscription. Per volume, cloth, $5.00; leather, $6.00; half-morocco, $7.00. Philadelphia and New York: Lea Brothers & Co., Publishers.

